# A Narrative Review of Commercial Platforms Offering Tracking of Heart Rate Variability in Corporate Employees to Detect and Manage Stress

**DOI:** 10.3390/jcdd10040141

**Published:** 2023-03-27

**Authors:** Craig S. McLachlan, Hang Truong

**Affiliations:** 1 Centre for Healthy Futures, Torrens University Australia, Sydney 2010, Australia; 2 Newcastle Business School, College of Human and Social Futures, University of Newcastle, Callaghan 2308, Australia; 3 Business School, Torrens University Australia, Sydney 2000, Australia; 4 Department of International Management Science, Thai Nguyen University of Information and Communication Technology, Thai Nguyen City 250000, Vietnam

**Keywords:** heart rate variability, employee, stress, cooperate, COVID

## Abstract

The COVID-19 pandemic has resulted in employees being at risk of significant stress. There is increased interest by employers to offer employees stress monitoring via third party commercial sensor-based devices. These devices assess physiological parameters such as heart rate variability and are marketed as an indirect measure of the cardiac autonomic nervous system. Stress is correlated with an increase in sympathetic nervous activity that may be associated with an acute or chronic stress response. Interestingly, recent studies have shown that individuals affected with COVID will have some residual autonomic dysfunction that will likely render it difficult to track both stress and stress reduction using heart rate variability. The aims of the present study are to explore web and blog information using five operational commercial technology solution platforms that offer heart rate variability for stress detection. Across five platforms we found a number that combined HRV with other biometrics to assess stress. The type of stress being measured was not defined. Importantly, no company considered cardiac autonomic dysfunction because of post-COVID infection and only one other company mentioned other factors affecting the cardiac autonomic nervous system and how this may impact HRV accuracy. All companies suggested they could only assess associations with stress and were careful not to claim HRV could diagnosis stress. We recommend that managers think carefully about whether HRV is accurate enough for their employees to manage their stress during COVID.

## 1. Introduction

### 1.1. Background

In the present study we wish to determine whether commercial heart rate variability (HRV) solutions are relevant to workplace organizations that are interested in improving employee stress and wellbeing. We are additionally interested in whether heart rate variability has been considered by commercial companies that monitor employee stress in the context of COVID conditions. The risk of contracting COVID-19 infection remains high for the working population, particularly in shared office workplaces [1]. This means a significant proportion of post-COVID-infected individuals will have an affected or damaged [2] cardiac autonomic nervous system. This has implications for measuring physiological heart rate variability responses that may be persistently altered chronically due to a known or unknown COVID-19 infection [3,4]. This being the case we question the utility of HRV measurements to detect stress in employees with a prior COVID infection for stress monitoring in the workplace.

Heart rate variability changes have been shown to be associated with both acute and chronic stress in laboratory settings and in occupational research [5,6]. Employee management of organizational stress is essential for preventing problems with mental health and wellbeing [7]. In summary, this paper seeks to understand and evaluate whether sensor device companies monitoring stress via HRV in the workplace also consider interactions with COVID-19 infection. To consider whether solution providers consider the impact of COVID on HRV, we will assess informatic web content and associated blogs of five well-known commercial companies offering HRV monitoring solutions to large scale enterprises for employee stress management.

A refined definition of HRV, for the purpose of this paper, is a series of derived parameters that are usually obtained from continuous heart rate changes over a short period to reflect systemic autonomic nervous system activity [8]. Continuous changes in the time intervals between consecutive heart beats are known as interbeat intervals and these are influenced by cardiac nerve activity innervating the sinoatrial node of the heart [8]. There are two types of autonomic nerves innervating the cardiac tissues, sympathetic and parasympathetic neural activity. Cardiac autonomic nervous activity is influenced by the balance of the sympathetic and parasympathetic nervous system; either nerve pathway may be influenced by an increase (stimulation) or a decrease (withdrawal) in nerve activity. An increase in sympathetic activity may increase heart rate and stimulation of the parasympathetic vagal nerve may slow the heart rate [9,10]. However, the rate of activity of either nerve pathway is non-linear to heart rate changes [11,12]. The sinus node regulates heart rate directly. Alterations in beat-to-beat analysis (from one heartbeat to the next) have some variability; this is due to resultant subtle changes in beat-to-beat interval oscillations around its mean value. This variability in heartbeat cycles permit measures of HRV (e.g., its derived parameters of autonomic function) from a continuous electrocardiogram or pulse sensor system [11]. In summary, HRV parameters allow estimates of the cardiac nervous system [12]. HRV is not a pure measurement of the cardiac nervous system, nor does it approximate changes in heart rate (HR). Beyond the heart the sympathetic nervous system has a significant role in the regulation of many physiological systems such as blood pressure, sweating and pupil activity.

HRV monitoring devices have gained consumer and commercial interest for their purported ability to detect stress and to allow for individual self-assessment [13]. HRV-based platforms, aligned to corporate managers with wellness portfolios, would allow assessment of aggregated biometrics, HRV and stress reports across functional teams. Interestingly, HRV is not a specific measurement. HRV changes are influenced by many factors including environment, lifestyle, activity, circadian rhythms, physiological and cardiovascular feedback loops, cardiovascular disease, health, medication, sleep duration, depression and stress-related events [14,15].

For the employee, stress is likely not limited to working hours. Stress responses may be amplified after work hours [16]. Alternatively, stress may be related to home factors as opposed to work-orientated time periods or related to both work and outside work hours. Indeed, COVID work from home conditions have also blurred the lines between work and home life [17]. Whether commercial stress monitoring providers of HRV biometrics consider stress events across the waking day is not known.

Interestingly there is also a metric trend to understand the duration of sleep with many HRV tracking systems such as Fitbit [18]. Sleep duration is important and has been shown to interact with other emotional factors and environmental stress responses [19]. Reduced sleep can affect stress levels directly. Reduced sleep can also affect HRV, and stress can also affect HRV as per the focus of this paper.

It would be interesting to also understand whether commercial HRV platforms that allow companies to track work factors that contribute to employee stress. It is likely that several HRV solutions for enterprise have historically been based on personal fitness and training and then expanded or pivoted to include corporate services such as stress monitoring (where more open data sharing may be part of a platform solution). This of course will create some ethical considerations when sharing private personal data such as HRV and stress assessment scores [20].

Digital biometric and non-biometric platform solutions have scaled to both communities and corporations during the COVID-19 pandemic. There is appreciation that stress management is important for productivity and employee engagement in the firm [21]. The practical implication of platform HRV data to predict individual stress responses has been based on many years of normative data without the background of COVID. We speculate it would be unlikely that companies would adjust prediction algorithms for stress detection based on COVID infection status and physiological responses post COVID infection.

A significant proportion of those infected with COVID will go on to have long COVID and/or persistent sustained levels of immune dysfunction which is pro-inflammatory and likely contributes to depression, anxiety, higher levels of stress and mental fatigue [22,23]. The fundamental question is whether a digital health technology solution is helpful in identifying and modifying stress behaviors and cognitions that may have become blunted during post-COVID infections or stress related to pandemic conditions. There exists a risk when management believes that HRV technology is acutely monitoring and correcting worker stress levels when the paradigmatic belief may be incorrect.

### 1.2. Literature Review

During the ongoing COVID-19 pandemic, several fitness apps that have measured heart rate and HRV for exercise fitness tracking and recovery [24] have expanded to also include corporate stress and wellness monitoring. However only a handful have utilized HRV as a biomarker for stress detection and or monitoring [25]. An exploration of the literature makes it known that there are several published studies using HRV to evaluate occupational stress [26]. However, it remains unknown firstly whether the implementation and sustainability of a mental health app solution for an organization using biometric data tracking will accurately monitor or predict stress responses and secondly whether these physiological parameters of stress can be restored by the individual [27,28].

Employee stress and burnout during the COVID-19 pandemic has escalated across businesses, affecting human resource productivity and staff turnover. Chronic stress and burnout have traditionally been defined as an imbalance between job demands and an employee’s mental and resilience; however, COVID may erode resilience and amplify stress responses (both acute and chronic). Burnout has been associated with stress and negative self-cognitions, where an employee may feel detached from an organization, lack self-interest and self-motivation and suffer reduced self-efficacy [29]. Many have criticized stress definitions as being vague, and the same can be said of definitions of burnout. This is important, as definitions of stress need some context for platform solutions to manage workplace stress [30]. Indeed, it will be interesting to see in our HRV platform analysis of providers whether they generically define stress via a vague label or provide a contextual definition relevant to the self or workplace stress or both.

As many employees continue to work from home [31], organizational workload thresholds are exceeded and work life balance is distorted for employees, which affects sleep duration and quality. Many apps track sleep quality, duration and sleep patterns. During COVID there have been many studies suggesting a decrease in the duration of sleep to 6 h or less a night; this lack of sleep can contribute to increased levels of stress or heightened stress responses [32]. Changes to sleep duration and patterns have been reported during acute COVID-19 infection [33] or post recovery [34]. HRV and sleep [35] are also associated, where poor sleep quality across many studies has been shown to adversely affect HRV parameters [36].

There has been interest in the ability of the individual to recover from stress by monitoring physiological states (assessed by biometric data) [37]. However, it is also understood that such activities will not address sleep, stressful events outside of work or post-COVID infection changes. Several reviews and opinions exist in the literature on corporate health programs and whether they have resulted in behavioral changes that lead to effective physiological changes. There is the false notion that improvement in perceived stress may lead to behavioral change and physiological return to baseline states [38]. Perceived stress is one’s self-awareness of the presence of stress related to an environmental or external influence. Here a stress event exceeds the individual’s cognitive resilience to stress and triggers a physiological and psychological stress response. For the worker in a corporate enterprise the stress response may be acute, chronic or acute on chronic [39,40]. The concern with perceived stress leading to psychological stress is that it can cause ongoing health and wellbeing issues such as burnout, depression and anxiety [40].

From an organizational perspective, HRV and HR tracking are used to manage an employee’s perceived stress levels. In doing so it offers a potentially cost-effective sustainable solution as a “plug and play” approach. However, from a sustainability perspective, organizations will ideally wish to understand whether an HRV-based platform can inform stress monitoring or provide accurate stress detection that can be addressed either by the individual or the firm. Currently, there is no research with respect to reviewing HRV stress monitoring for employee stress reduction and no knowledge whether there is additional consideration for the effects of COVID infection.

**Figure 1:**
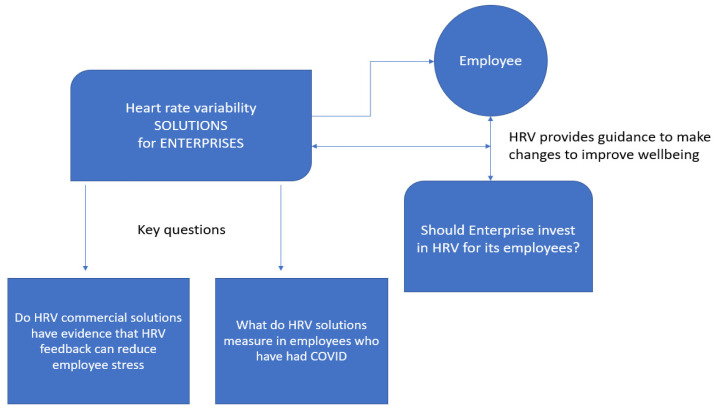
Key theoretical concepts and observational gaps emerging from the literature review: (i) accuracy and sustainability of a HRV tracking solution for identifying employee stress; (ii) it remains unknown whether HRV solutions to monitor stress are accurate in employees who have had COVID-19.

Our aims highlight the gaps in knowledge surrounding HRV monitoring devices with respect to detection of stress in employees under COVID conditions (see Figure 1).

The broad aims are to explore HRV commercial tracking solutions for stress monitoring and alerting across a sample of relevant commercial service providers. In doing so the following specific aims will be addressed:

i. To assess background scientific evaluation of these commercial HRV tracking platforms in the literature. Specifically, whether there has been literature that evaluated the ability to detect stress with these commercial providers has been provided.
ii. To assess whether HRV commercial services provides guidance around post-COVID syndromes and ability to detect stress.
iii. To assess the time periods for self-monitoring that commercial providers recommend.
iv. To assess what claims have been made about the accuracy of using an HRV stress device for employee stress detection or management.
v. To determine if stress has been defined by each providing company we have assessed.

**Hypothesis** **1.** *Companies that are offering platform HRV monitoring for stress management to corporations have not considered the impact of COVID infection on biometric stress detection in individual employees*.

**Hypothesis** **2.** *HRV platforms that use HRV to detect stress are not consistent across platform solutions and therefore are of questionable reliability*.

**Hypothesis** **3.** *There is a lack of scientific validation for such technologies using HRV platforms to detect stress in workers in the literature*.

## 2. Materials and Methods

### Inclusion of HRV Platform Companies for Analysis

**Figure 2:**
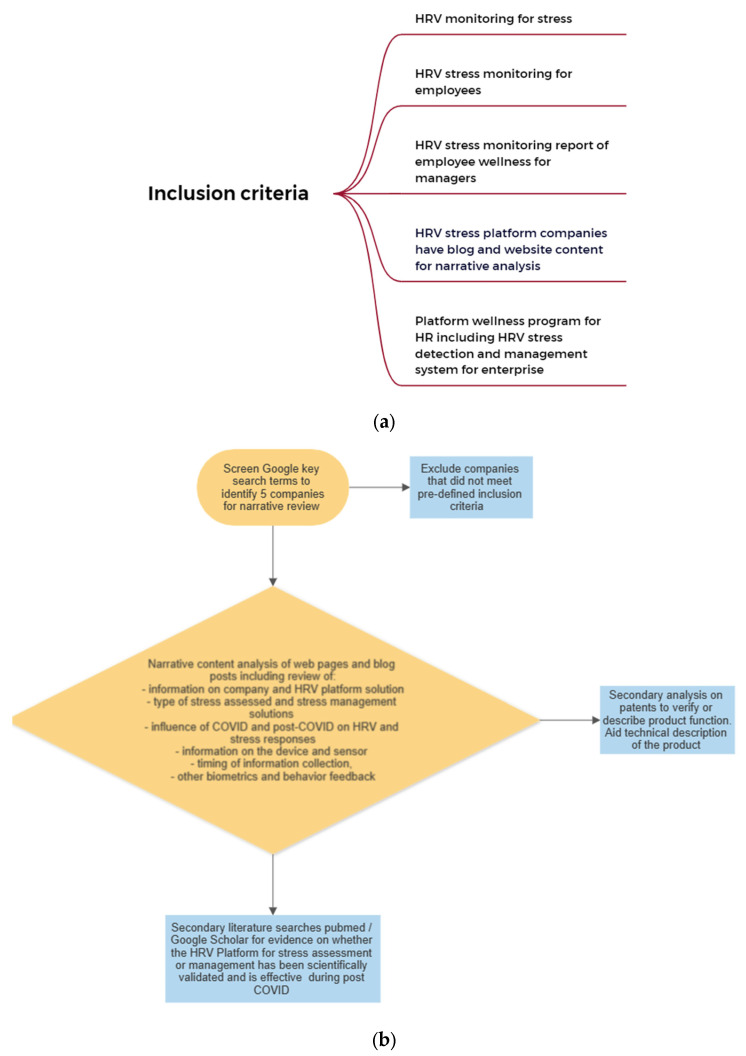
(**a**) Summary of inclusion criteria for selecting HRV stress platforms for analysis. (**b**) Flow diagram of the steps in identification of the 5 companies and the sequence of primary and secondary analysis of HRV platform product offerings. Analysis addressed the aims and hypothesis. Please note that companies were searched until 5 were identified for narrative analysis; no analysis was captured of the number of companies not included. Scientific literature searches were to identify any articles that related to the selected company HRV platforms. Literature identified or not identified was used to verify the presence or absence of any scientific evaluation of these HRV platforms to assess and manage stress.

We searched Google for companies providing stress screening using HRV as part of their offering solution. We included companies identified up until the 5 August 2022. Key combinational word search terms included “stress”; “COVID”; “heart rate variability”; “HRV”; “enterprise”; “company”; “employee”; and “biometrics”. A commercial HRV platform solution had to meet the following conditions: (i) had a considerable population of corporate consumer end-users, (ii) the platform targeted employees within enterprises and used HRV as a primary parameter to assess individual stress levels, (iii) had considered stress as a modifiable factor (iv) had traction in providing physiological monitoring, wellness or biometric solutions as a mature company offering to large-scale enterprises, (v) the company website had blog posts and other information relevant for narrative content analysis. While there are many HRV solutions for individual stress tracking in the marketplace, many are not targeted as specific enterprise wellbeing solutions. Inclusion also required both worker and management metric reporting. We have only included companies that both allow for such tracking of the individual worker and are targeted to enterprise (Figure 2a).

HRV stress tracking platforms were randomly assessed until saturation with five platforms identified for analysis.

For each platform, a narrative analysis was conducted with their web pages and associated blog posts with the following factors assessed (Figure 2b): (i) whether HRV (individually or as a component of the offering) can be used to detect stress and whether there is evidence for the type of stress detected; (ii) whether stress detection was considered with respect to the influence of COVID infection on the autonomic nervous system (Hypothesis 1); (iii) the level of discussion around COVID and need for stress monitoring for employees (Hypothesis 1); (iv) whether there has been a scientific evaluation of each HRV platform with respect to effectiveness as a stress reduction programs for corporations (Hypothesis 2 and 3); and (v) the timing and amount of the stress monitoring across platforms (Hypothesis 2). The narrative analysis conducted was not amenable to a systematic review protocol framework. Content analysis was performed on blog posts and websites associated with each of the identified companies meeting our criteria. Narrative reviews are the most common type of articles in medical literature; we addressed the recommendations of the SANRA scale to ensure the quality of our framework and methodology for our outputs [41].

## 3. Results

The results support the descriptive analysis of the platforms examined below when exploring the web content and blogs related to each platform. Additionally patent claims and descriptions of the invention within may be examined to clarify blog posts. The level of confidence of stress detection was assessed as well with and without COVID considerations (Hypothesis 1); any published literature to support claims for evidence-based service provision during COVID infection is also provided (Hypothesis 1). Additionally, narrative content is explored, examining whether stress detection is multi-focused (Hypothesis 2).

Descriptive analysis of the key elements contained within each of the five platforms is provided below.

i. Total Brain (San Francisco, CA, USA) is an Australian listed company with head offices in the USA and is focused on improving corporation employee brain health with a particular emphasis on stress identification and early management using relaxation techniques [42]. Total brain has used a platform-approach offering to corporations for their employees. The company recognizes that life events may trigger stress in an employee at any point in time. Hence, continued assessment is recommended for employees. The Total Brain platform focuses on self-monitoring stress via HRV (providing an automated stress reference score based on built-in algorithms) and self-care apps for the individual employee that allow for interventions such as resonate breathing. Individual data within an organization can be aggregated for management and team leaders to review.

The self-care concept means that individuals can detect stressful events and undertake activities to reduce immediate stress events and autonomic activation. For example, immediate threats in a working environment may trigger a fight-or-flight response, associated with increased autonomic sympathetic activity. If increased physiological stress as opposed to perceived stress is detected by the individual, that individual may be guided by an app to undertake paced breathing and mediation. The focus on resonate breathing is emphasized.

Interestingly HRV is measured via a phone’s camera, a photoplethysmography approach. Total Brain transforms the user’s mobile phone camera into a pulse rate measurement device. The light from the flash of a phone’s camera permits infrared imaging of the blood vessel pulse in an individual’s fingertip and from this HRV is calculated based on pulse variation. The HRV parameter reported upon is RMSSD (Root Mean Square of Successive Differences). This RMSSD index is then compared to Total Brain’s normative database to provide a score from 1 to 100 that reflects a user’s current level of calm and focus compared to others of the same age. The Total Brain platform in summary centers on the employee to self-monitor and rapidly restore any physiological stress detected.

The app also allows for self-monitoring of mental health conditions and tracking mood and emotion. The use of self-reported mood is coupled with HRV to provide a more informed stress feedback score. While COVID is recognized to increase stress, no mention of other COVID effects on the autonomic nervous system and HRV were mentioned in blog posts or scientific publications.

ii. Fitbit (San Francisco, CA, USA) is an American consumer fitness biometrics company. Fitbit at the time of this paper is releasing a wrist watch sensor based system called Pulse that incorporates HRV analysis, HR capture via photoplethysmographic (PPG) sensors (disclosed in patent US20150201853A1) and possibly other biometrics to monitor stress levels for specific life events. A review of patents by Fitbit to better understand their interest in stress was performed; the use of their technology dates to 2010 when a patent was granted for a stress detecting system based on sensors to measure HR and derive HRV biometrics. There are also patents granted for using wearable sensor devices that include prompting the user, via an interface, to perform a meditation exercise (e.g., guided by a respiration target) [43], and may be guided by a biometric improvement, such as HR or HRV parameters [44]. It is also recognized that the ability to measure electrodermal activity with Fitbit technology is now available on some of their wearable models for detecting additional stress responses [45].

Biometric analysis of HRV and pulse rate is aimed at increasing self-awareness of stress in employees. AI algorithms are built into the app to guide employees to recognise and reduce stress [46]. There is also the option for counselling or coaching if the individual needs additional support for stress management. Fitbit also suggests that triggers of stress can be integrated with standard time and date (calendar) technology and GPS to self-reflect on the location and time of the stress event. Interestingly, Fitbit also mentions the need to promote financial fitness in corporate employees as this was suggested to be an important stressor. The company had beta tested the app in corporate employees and demonstrated a 14 percent decrease in anxiety, 10 percent decrease in stress and 8 percent decrease in burnout among employees as well as a modest positive effect on resilience [47]. Fitbit describes the need for more individuals to self-monitor and track stress due to the COVID-19 pandemic resulting in excess stress.

There is no evidence to demonstrate that corporate Fitbit technology for employees has been tested in employees with prior COVID infection and still permits accurate associations with stress responses.

iii. Komodo Technologies, Inc. (Winnipeg, MB, Canada) focuses on solutions involving HRV and HR analysis [48]. Kondo technologies developed a Smart wearable compression sleeve with biosensors. Specifically integrated with smart fabric and sensors, it allows for health and fitness analysis. The company has also developed a corporate wellness solution for employees, the AIO Health Bar [49]. The AIO Health Bar has been described by the Komodo as interactive health kiosk that conducts stress tests for companies and other interested community groups. The health kiosk is a stand-alone unit paced in a common office area that is for stress testing only using HRV and HR and from which each employee receives a stress report. Interestingly, Komodo Technologies also understands that random time of day measurements may not mean much to assess stress, so it has timed daily check-ins where stress levels are suggested as being measured using HRV and HR. Examples of event periods include what is termed “morning readiness”, before and after work, post-meal, and post-workout. guided by an aggregated stress score based on activity, sleep, and heart activity [50]. There is no evidence that this technology has been evaluated in COVID-positive employees.
iv. WHOOP (Boston, MA, USA) is a biometric platform where one of the components is HRV. These biometrics collected via individual biofeedback help support or motivate personal training and modifying behaviors related to lifestyle. The company recognizes that there are many factors that may influence stress, including exercise training factors, lifestyles factors (including alcohol intake, relaxation, sleep quality and duration) [51] and biological factors (including aging, gender, genetics and disease processes). Hence not all of these will be modifiable factors that can be addressed to positively influence HRV. WHOOP collects HRV data via the WHOOP Strap (WHOOP, Inc., Boston, MA, USA), a validated, wrist-worn biometric device. Biometric measures of sleep, resting heart rate (RHR), heart rate variability (HRV) and respiratory rate are measured via the WHOOP strap [52]. The WHOOP website suggests that HRV is meant to be optimized at a personal level to attain improvement in HRV indexes through modifiable lifestyle or training factors. Whoop is careful not to suggest that HRV can monitor stress in the absence of considering other factors or lifestyle interventions.

In addition, WHOOP has a corporate stress monitoring and management solution, called WHOOP unite. The aim according to the website is to target employees in teams and to monitor potential changes in aggregated physiological stress biometrics [53]. The aim of managers is to review summary data insights to understand changes in team biometrics and to encourage employees to self-improve their mental health and physical activity. This can be either directly and/or via the use of WHOOP coaching. Targets for self-improvement include personal optimization of factors affecting stress, burnout and sleep quality. The website suggests any positive changes at a team level are likely to lead to increased resilience within the firm [53]. Stress is believed to influence staff turnover due to the development of burnout. WHOOP has written blog posts about its concern for burnout in organizations during the COVID pandemic. The use of HRV with other biometrics is to monitor environmental factors contributing to presumed physiological stress responses. It is difficult to suggest that these factors are internal or external to an organization; however, the end outcome includes potential factors that could negatively impact sleep duration and thereby affect HRV, HR and respiratory rate.

WHOOP reports that sustained stress can lead to burnout, depression and anxiety during COVID [46]. However, there has been no published validation of the technology to detect stress in post-COVID subjects.

v. Firstbeat Life (Jyvaskyla, Finland) is a platform for analyzing HRV data from individuals [54]. Firstbeat Life have three-tiered services: for the individual, monitoring aggregated data for corporations and services for wellness professionals. Firstbeat Life as a heart rate variability reporting platform for enterprise suggests that Firstbeat Life will allow their technology to address and promote company-wide health and wellness [55]. Firstbeat Life focuses on the use of HRV analysis to identify individuals’ potential stress levels, and in being self-reactive to these stress levels, suggest it is possible to achieve a happier and more resilient workforce. Summary metrics can be shared with management or teams. There are several categories of scientific publications (that have undergone peer review) on the Firstbeat website. However, there is a lack of published evidence that the use of Firstbeat Life using HRV can achieve a happier and more resilient workforce, and no studies have shown that their technology in post-COVID-infected individuals within an enterprise setting can reduce stress using HRV. Interestingly, Firstbeat Life have conducted studies during the COVID pandemic (April to October 2020) in work from home employees, with a large metric of pooled individuals accounting for 26,000 measurement days. A total of 90 percent of the participants were based in Finland [56]. The finding was that stress-related measures of HRV declined, while active movement also decreased; they explained the improvement in HRV recovery levels as being due to a modest average increase in daily sleep of 14 min.

The Firstbeat Life system produces a report for individuals that provides stress-related visualizations, and individual users use that information to make relevant behavioral changes that presumably reduce their stress levels. However, it should be noted that previous evaluations of workers using the technology did not see any improvement in wellness based on subjective reports. Additionally, there has been no published validation of the technology to detect stress in post-COVID subjects.

The types of HRV stress detection and evidence for stress associations across the different companies are summarized in Table 1.

## 4. Discussion and Conclusions

Our content and literature analysis of commercial providers supplying corporate HRV stress-tracking systems reveals a lack of published studies and non-published blog information regarding the impact of COVID-19 infection on cardiac autonomic dysregulation. Our research findings support the original Hypotheses 1–3. Autonomic nervous system changes post-COVID infection have implications regarding the ability to functionally track stress associations based on changes in HRV. COVID infection that contributes to a dysfunctional autonomic nervous system in a large proportion of infected and post-infected individuals will also affect previous baseline cardiac autonomic states [57]. Variable baseline states of the autonomic nervous system would be problematic for companies wanting both to track and allow employees to accurately assess and manage their stress levels. Changes in HRV and HR after vaccination returned to baseline after a few days [58]. While companies have been quick to suggest their technologies may be useful for remote monitoring of acute COVID infections, they have not mentioned the implications of persistent physiological changes for stress monitoring.

Where COVID was addressed in company blogs or webpages, it was to show that their technology had contributed to fitness during COVID or had some limited role in diagnosing acute COVID infection [59] and tracking vaccine responses [60].

Interestingly no companies we are aware of have explored the effects of long COVID on work stress using HRV. Some companies also used external COVID data to show an increase in worker burnout, suggesting a link between stress and burnout. From a marketing perspective this was most likely to promote the uptake of the technology to corporate organizations to protect their employees from burnout by managing and tracking their stress using HRV. However, as mentioned in the introduction stress does not always lead to burnout [61,62] and stress can have a positive influence on learning and worker productivity [63]. We also noted that the generic term stress was used by all providers and not defined but the majority did relate stress to worker burnout. Total Brain, however, did try to also understand how stress may affect emotional and cognitive processing and relate this to HRV tracking.

All commercial providers were careful not to market their technologies as having an ability to diagnose stress. Currently there are no clinically approved technologies that support this claim. We also noted that companies did suggest that HRV was not static and needed to be measured at several time points to derive a global stress score. However, we also noted that one company offered a stationary unit for employees to measure stress levels in an office setting. We speculate that frequent visits to a fixed HRV recording device in an office environment will have privacy issues and may also add to stress [64,65].

Historically the larger companies we have analyzed have held a strong market segment for measuring HRV in athletic fitness such as WHOOP and Fitbit. There is evidence that a lack of fitness will affect cardiac autonomic recovery and HRV parameters and stress responses [66,67].

We also evaluated if companies have published any scientific studies that would support the use of their technology to assess stress in an organizational framework. We found that Firstbeat Life reported on one study showing an ability to reduce HRV indices while working from home by increasing moderate amounts of sleep per day, despite daily activity decreasing [56]. It is unknown if this is related to sleep, a real reduction in stress or something else. These data from Firstbeat Life were reported at the start of the pandemic, where COVID infections would have affected less of the global population. Another company, Fitbit, suggested a 10 percent reduction in stress in workers using their technology in a testbed that was not published. There are no published studies we are aware of using HRV to modify stress responses post COVID infection. Importantly, several companies also measured biometric sleep duration in addition to HRV to provide a global score of stress. It was also recognized by companies that measuring HRV more than once per day is required to reflect changes in physiological stress [68].

An important outcome from our findings is the need for management to consider further the utility of an HRV biometric solution for employee stress management before investing in this technology. Managers that address wellbeing needs of the organization need to understand what type of stress they wish to manage for the individual worker and the time of day (e.g., work stress factors, worker stress factors during work or both work and home) [69]. Wellbeing program decision makers also need to understand whether HRV and other associated biometrics are a good proxy estimate of the stress type they are interested in for employees. Additionally, managers should ascertain whether a stress alert or stress metric can be quantified for the organization. Any corporate wellbeing program ideally has a clear understanding that these HRV systems are not diagnostic. In addition, there may be significant noise from prior COVID infection (even if the employee is recovered) masking stress regulatory responses assessed by HRV. There are other factors related to HRV responses such as age, fitness, chronic disease and pathological processes that influence the autonomic nervous system negatively such as inflammation or hypertension [70,71].

**Figure 3:**
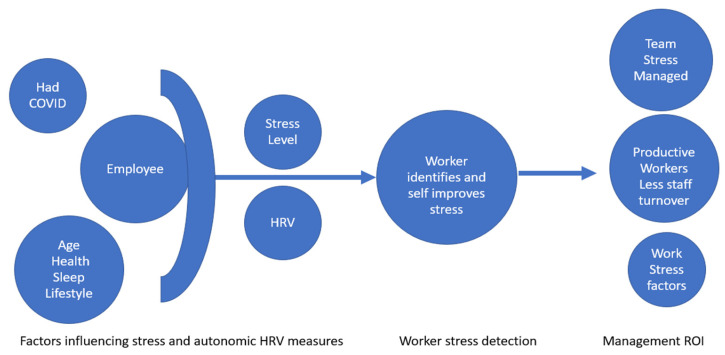
Conceptual model on key factors that would serve corporations tracking stress with HRV and modifying factors that may create excess noise in system. Return on Investment (ROI).

Our findings create a pragmatic framework model to consider whether HRV measurement of employees (around stress monitoring and reduction) aligns with ongoing COVID conditions; that is whether an HRV biometric solution is useful to monitor and inform both the employee and management (see Figure 3).

Figure 3 is a conceptual model showing factors that influence interest from the management side to assess employee stress levels. On the far left side, modifying factors are included that could decrease accuracy further when using HRV to assess individual employee stress responses. For example, an employee will have demographic, pre-existing differences and changes in health, lifestyle and fitness, all of which can influence both stress and the cardiac autonomic nervous system. As alluded to HRV is derived from continuous changes in the cardiac autonomic nervous system and acute changes in HRV may identify stress responses or changes in baseline HRV. Pre-existing COVID we identified would likely add significant noise to HRV and possibly stress responses. Following any identified stress from HRV changes, ideally, the worker would need to self-adjust lifestyle factors or use bio-feedback relaxation to improve their HRV. Post-COVID changes to cardiac autonomic control would be immune to lifestyle changes. Constant monitoring and daily adjustment are thought to prevent progression of stress. Apart from employees’ self-help, a provider may offer coaching or counselling services to aid stress management. Importantly, a firm will wish to justify costs for any stress management system invested in a corporate wellness program. Managers, for example, will track global stress and want to identify any stressors within the organization that contribute to employee stress levels. Indeed, reoccurring work stressors would be identified by employees. Management can review the stress of the organization across the aggregated stress of teams and align stress reporting to productivity tracking. In summary, stress reduction at an employee level is self-actionable and should be rapid and result in sustained lower stress levels. However, whether HRV can track stress in previously COVID-infected individuals or in individuals with chronic health conditions that affect the autonomic nervous system remains a questionable notion.

## 5. Limitations and Future Studies

We have focused on assessing HRV strategies around current large-scale technologies aimed at providing on-demand employee biometric HRV measurements to detect and manage mental stress (in and outside the workplace). Employee self-assessment and self-management are drivers for wellness programs during the COVID pandemic. We have used an informatics approach to assess the role of HRV in the provision of stress monitoring as an organizational solution by exploring commercial provider web and blog content. We have shown that there is an absence of planning and systems in place to support HRV measurement accuracy if an employee were to have post-COVID autonomic dysfunction or abnormal heightened or blunted responses to stress. There is likely a lack of mechanisms to share data back to management to track stressors specific to an organization because the focus is on the self-control of stress by the individual. In summary, stress is not linked to identifiable factors within the workplace.

We have provided a theoretical framework model of the risks for management to engage in HRV platform monitoring of stress in the workplace. We have also provided factors that may be affecting the autonomic nervous system adversely; these same factors would interact and interfere with the stress responses being measured on an HRV platform. We recognize that the firms we have reviewed also include other tools and measures alongside HRV to try and improve the stress score but many of these measures are co-dependent—meaning, for example, sleep itself can also affect HRV and sleep can be influenced by stress [72,73,74,75]. We recommend that future research addresses the need to better understand the dual effects of health and COVID infections on individual stress in and out of the workplace and to understand the causation of stress influences.

In conclusion, HRV stress platforms for employee self-tracking of stress are likely not designed to support management optimizing of stress if HRV is at best a quasi-estimate with known error or the influence of non-stress factors. Interestingly, many of the tested platforms are measuring HRV at random times of day across the adult lifespan. This makes longitudinal tracking problematic, where both time of day, duration of recording and age of the participant can have variable results on HRV independent of COVID status [76,77]. There are many factors that can affect sustainability of stress-management systems implemented as part of a wellness program for an enterprise [78]. From the perspective of our study, firstly, the sustainability of a stress-monitoring platform solution is questioned when the type of stress being measured has variable meanings. Secondly, that stress can have a positive impact on a worker’s productivity and does not always need to be managed. Thirdly, there is no evidence that HRV can accurately diagnose stress levels from a clinical perspective; rather physiological changes that may be associated with stress are monitored. Finally, HRV would be a problematic measure where the cardiac autonomic nervous system is dysfunctional in employees with some chronic health conditions or in the large proportion that has recovered from COVID-19 infection. We also recommend to HRV providers that they assess the impact of COVID infection and other known health conditions on HRV such that they may be able to develop more accurate prediction models of stress events. A lack of communication from providers of stress-monitoring services to corporations around the impact of COVID could have negative marketing consequences on any positive utility of self-monitoring and tracking of HRV. Possibly, HRV is a victim of its own success in its ability to associate positively with many health conditions, moods and emotional self-cognitions.

## Figures and Tables

**Table 1:** A comparison of HRV platforms using open access information (web and scientific published literature). Here we provided the evidence and function of each HRV platform with respect to stress engagement for each commercial company assessed. Heart rate (HR); respiratory Rate (RR).

Company	Type of Stress Output	Type of Stress Is Defined	Multiple Times of Day Measures Encouraged	Published Scientific Evidence to Show Effective in Reducing Corporate Stress	HRV Only or Other Biometrics
Total Brain	Stress	No	Yes	No	HRV
Fitbit	Stress score	No	Yes	No	HRV, HR, Sleep, Electrodermal
Komodo	Stress report	No	Limited to work hours	No	HRV and HR
WHOOP	Stress score	No	Yes	No	HRV, HR, RR, Sleep
Firstbeat	stress-related visualizations	No	Yes	No	HRV

## Data Availability

All data is contained within the manuscript.
